# Diets for patients with chronic kidney disease, should we reconsider?

**DOI:** 10.1186/s12882-016-0283-x

**Published:** 2016-07-11

**Authors:** William E. Mitch, Giuseppe Remuzzi

**Affiliations:** Department of Medicine, Nephrology Division, Selzman Institute for Kidney Health, Baylor College of Medicine, M/S: BCM 395, One Baylor Plaza, ABBR R703, Houston, TX 77030 USA; IRCCS - Istituto di Ricerche Farmacologiche Mario Negri, Bergamo, Italy; Department of Medicine, Unit of Nephrology and Dialysis, Azienda Socio Sanitaria Territoriale, Papa Giovanni XXIII, Bergamo, Italy; Department of Biomedical and Clinical Sciences, L. Sacco, University of Milan, Milan, Italy

## Abstract

Here we revisit how dietary factors could affect the treatment of patients with complications of chronic kidney disease (CKD), bringing to the attention of the reader the most recent developments in the field. We will briefly discuss five CKD-induced complications that are substantially improved by dietary manipulation: 1) metabolic acidosis and the progression of CKD; 2) improving the diet to take advantage of the benefits of angiotensin converting enzyme inhibitors (ACEi) on slowing the progression of CKD; 3) the diet and mineral bone disorders in CKD; 4) the safety of nutritional methods utilizing dietary protein restriction; and 5) evidence that new strategies can treat the loss of lean body mass that is commonly present in patients with CKD.

## Background

When we published a Commentary on how dietary factors could affect the treatment of patients with complications of chronic kidney disease (CKD) in 2004, we relied heavily on results from older publications [1]. There were two reasons for this decision: firstly, clinicians and investigators working on this topic were still reeling from the negative conclusion of the Modification of Diet in Renal Disease trial (MDRD), namely that dietary modification exerted only a minor impact on the progression of CKD [2]. Secondly, there were too few hypotheses addressing how dietary manipulation could affect the development of CKD and its complications [1]. We now revisit this topic because new insights have identified how dietary factors can overcome the development of CKD and its complications, including the progression of CKD. Notably, these insights can be largely traced to results obtained during rigorous studies of patients with CKD rather than intensive investigations of animal models, indicating there is clinical relevance to the reports. To illustrate why we are keen to bring these developments to the reader’s attention, we will briefly discuss in this article five CKD-induced complications that are substantially improved by dietary manipulation.

## Dietary changes that correct metabolic acidosis can suppress complications of CKD

The genesis and treatment of metabolic acidosis has long been a favorite teaching exercise for nephrologists [3]. This interest arises in large part because this complication of CKD causes substantial loss of muscle mass [4–7]. The scope of topics regarding the treatment of metabolic acidosis has grown substantially because it has been reported that correction of metabolic acidosis by dietary manipulation can effectively suppress the accelerated loss of kidney function in patients with progressive CKD. This insight was first detected when it was found that the serum levels of bicarbonate were inversely associated with increases in serum creatinine in the calculation of estimated GFR (eGFR). For example, Shah et al., examined clinical chemistry results obtained from adults attending a University Medical Clinic over a 2 year time period [8]. In their study of 5422 patients with various kidney diseases, including diabetic nephropathy, 337 patients were found to have evidence of CKD progression as assessed by declining eGFR. In patients with serum bicarbonate concentrations ≤ 22 mEq/L, the hazard ratio for the progression of kidney disease was 1.54 (95 % confidence interval; [C.I.] 1.13 to 2.09) after adjustment for potential confounders compared to the reference group (serum bicarbonate levels, 25 to 26 mEq/L). Similar results were obtained using different definitions of the renal outcome (i.e., 30 % decline in eGFR or doubling of serum creatinine levels) [8].

In another analysis, values of dietary acid generation were obtained from the 24-h dietary recall questionnaire used in the National Health and Nutrition (NHANES III) Trial [9]. In the 1468 adults with CKD participating in this evaluation during 14.2 years, 311 participants developed ESRD. The risk of ESRD was increased in patients expressing higher levels of dietary acid generation yielding a relative hazard ratio of 3.04 (C.I.; 1.58 to 5.86) for patients with the greatest degree of acid production.

Identification of the associations between metabolic acidosis and the progression of CKD has understandably stimulated investigators to examine if progression of CKD could be suppressed by simply supplying alkali supplements. De Brito-Ashurst and colleagues evaluated this possibility in a controlled trial of 134 adult patients with metabolic acidosis (serum bicarbonate, 16–20 mM) and CKD (basal creatinine clearances (Ccr), 15 to 30 ml/min/1.73 m^2^) [6]. In that study, patients were randomly assigned to standard care or to administration of bicarbonate supplements; their Ccr levels were examined repeatedly over 2 years. The patients assigned to bicarbonate supplements experienced a slower decline in Ccr (−1.88 vs −5.93 ml/min/1.73 m^2^; *P* < 0.0001) and fewer of the patients treated with sodium bicarbonate developed ESRD (*P* < 0.001). The investigators also uncovered nutritional benefits of correcting acidosis in patients with CKD, including evidence for increasing protein stores. Other investigators have examined patients with hypertension, CKD and relatively preserved levels of eGFR (mean, 75 ml/min/1.73 m^2^) during a 5 year period [10]. Enrolled patients were treated with ACEi and then randomly assigned to receive sodium bicarbonate, an equimolar amount of sodium chloride or a placebo. The authors found that the rate of decline of eGFR (estimated from plasma cystatin C levels) was slower while eGFR values remained higher in the group of patients treated with oral supplements of sodium bicarbonate compared to results from patients treated with sodium chloride or placebo. These and other reports demonstrate that the correction of metabolic acidosis not only slows the rate of loss of kidney function but also can improve hormonal metabolism as well as values of protein turnover [11]. Although it is possible that measurements obtained in a larger clinical trial might uncover different results, these clinical measurements were collected in randomized, controlled trials and hence, they offer overwhelmingly relevant reasons for correcting metabolic acidosis in patients with CKD.

**Fig. 1:**
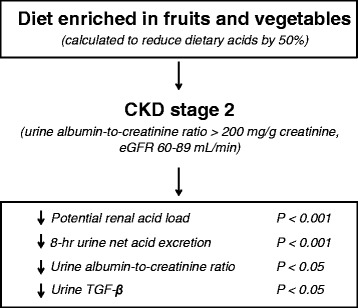
Diet enriched in fruits and vegetables in amounts was administered in order to reduce dietary acid load by 50 %. Patients with stage 2 CKD who received this dietary intervention for 30 days experienced significant improvements in the potential renal acid load, 8-h urine net acid excretion, urine albumin-to-creatinine ratio and urine TGF-β. Abbreviations: CKD, chronic kidney disease; eGFR, estimated glomerular filtration rate; TGF-β, transforming growth factor β. Results from Goraya et al. [16]

Do publications also document that dietary factors influence the relationship between metabolic acidosis and progression of CKD? The answer is yes because it is known that the catabolism of dietary proteins, especially if the diet supplied proteins of animal origin, is the principal source of acid generation [3]. When the kidney is challenged with acid, such as by eating an animal-source protein diet, it increases levels of hormones (i.e., angiotensin II, aldosterone, endothelin) that help it excrete the acid over short-term observations [12, 13]. However, in the long-term, high levels of these hormones worsen kidney function [14, 15]. Consequently, limiting proteins in the diet will reduce the generation of acid and should aid in the correction of metabolic acidosis. Indeed, there is evidence that protein restricted diets can also suppress the progression of CKD. For example, in 2012, these relationships were tested by Goraya and colleagues [16]. They reduced the daily amount of acid generated by simply increasing fruits and vegetables in the diet. This recommendation achieved the goal of reducing acid generation by 50 % [16]. The strategy used by the investigators simply amounted to increasing fruits and vegetables in the diet. The amount of added fruit and vegetables was based on adding what would be required for a 50 % reduction in daily acid generation. For most of the patients, this amounted to adding two to four cups of fruits and vegetables to their daily diets. In the clinical trial of the influence of adding these foods, hypertensive patients with stages 1 or 2 CKD (presumably due to hypertensive nephropathy) were treated with ACEi and outcome measures were documented [16]. The measures studied included a decrease in urinary markers of kidney damage, namely N-acetyl beta-D-glucosaminidase and TGF-β1. After 30 days, stage 2 CKD patients who were treated with either sodium bicarbonate supplements or changes in the diet designed to increase the content of fruit and vegetables had comparably positive responses estimated from decreased appearance of markers of kidney damage (Fig. 1). These positive results were obtained during studies of a small number of patients with relatively mild CKD but were sufficiently intriguing that the investigators were stimulated to undertake a larger trial [17]. In this second clinical trial, hypertensive patients with stage 4 CKD (eGFR of 15–29 ml/min/1.73 m^2^) and metabolic acidosis (i.e., total CO_2_ < 22 mM) were randomly assigned to either daily sodium bicarbonate supplements (1 mEq/kg/day) or to an increase in dietary fruits and vegetables that was sufficient to reduce dietary acid by 50 %. After 1 year of treatment, the total CO_2_ had increased to a similar extent (compared to baseline values) in both the sodium bicarbonate and fruits plus vegetables groups. Regarding kidney functional outcomes, the eGFR calculated from plasma cystatin C levels did not differ between the groups but indices of kidney injury were lower in patients treated with diets enriched in fruits and vegetables.

These reports are encouraging for several reasons. Firstly, there is evidence that simply supplying alkali to patients with CKD and metabolic acidosis will result in important clinical benefits that include slowing of progressive renal insufficiency and reversing the loss of protein stores. Secondly, the dose of sodium bicarbonate supplements or the amounts of dietary fruits and vegetables needed to block the development of metabolic acidosis can be simply assessed by measuring the serum bicarbonate (or total CO_2_). Thirdly, these outcomes should stimulate new hypotheses to design methods of correcting metabolic acidosis or creating other dietary strategies.

## Inadequate attention to the diet interferes with methods of slowing progression of CKD

Designing the optimal diet for patients with CKD is difficult because of interrelationships among different nutrients. For example, when the diet plan includes a reduction in dietary protein there must be a concomitant increase in the intake of other nutrients to maintain the requirements for calories. This is not the only problem since the requirement for calories is also influenced by CKD, because CKD results in the development of insulin resistance and eventual impairment of carbohydrates and lipids utilization [18, 19]. Moreover, designing meals must also include recommendations about the intake of ions and minerals. This is relevant because a high intake of protein is generally accompanied by an increase in dietary phosphates, salt, etc. This problem was highlighted when it was reported that unregulated amounts of dietary phosphates or sodium chloride will interfere with the ability of ACEi to suppress the progression of CKD. In one report, Zoccali and colleagues pointed out that changes in the levels of serum phosphate were positively correlated with values of serum creatinine in 331 patients with proteinuric nephropathies who were participating in the prospectively designed Ramipril Efficacy in Nephropathy (REIN) trial [20]. The investigators found that independently of treatment, patients with serum phosphate levels in the highest two quartiles (3.45–4.00 mg/dL and > 4.00 mg/dL) progressed significantly faster, reaching ESRD or a composite end-point of doubling of serum creatinine or ESRD when compared to results from patients with phosphate levels below the median (<3.45 mg/dL). Of note, the renoprotective effect of the ACEi, Ramipril, decreased as serum phosphate levels increased (P ≤ 0.008). This association persisted even after the results were adjusted for confounders such as measured GFR and urinary protein. The authors concluded that monitoring and reducing serum phosphate by changing the diet should be a principal goal in planning the diets of patients with CKD. We recognize that the admonition to control serum phosphorus by manipulating the diet can be very difficult because phosphates present in processed grocery products are only infrequently posted on the food labels [21]. For example, one test of the accuracy of food levels concluded that 44 % of the best-selling items in a grocery were found to contain phosphate additives, especially in prepared, frozen foods, package meat, bread, soup, etc. Not only is the phosphate content of many foods missing from the food content labels but there is evidence that this source of additional phosphates is clinically relevant: the analysis indicated that sample meals comprised mostly of foods that frequently contain phosphates amounted to 736 mg more phosphorus per day when compared to meals prepared with additive-free foods [21]. Notably, diets that are rich in animal-source protein are rich in phosphates [22]. Sherman and Mehta investigated the ratio of the phosphorus in meat, fish, and poultry meats and uncovered that the phosphorus content of 1 g of protein varied from 6.1 to 21.5 mg. Based on this information, it is not difficult to understand how restricting dietary protein can reduce the risk of adverse outcomes from high phosphate diets, including renal osteodystrophy, left ventricular hypertrophy and progression of CKD. In short, without careful dietary planning which includes the phosphate content of different foods, the benefits of ACEi therapy on progression of CKD and the prevention of renal bone disease will be eliminated.

Phosphates are not the only potentially harmful nutrients associated with protein rich foods. For example, sodium chloride is often added to processed foods to improve their “shelf life” plus the taste of the food product, specifically foods that are pre-prepared or are in the category of “fast foods”. As occurs with the high phosphate content of protein-rich foods, responses to a high salt diet can interfere with the ability of ACEi to suppress progression of CKD. A *post-hoc* analysis of the REIN trial showed that among 500 patients with proteinuric CKD receiving ACEi therapy, those with a high dietary sodium intake (i.e., >14 g of salt/day) had a three-fold higher risk for progression to ESRD compared to those assuming less than 7 g of salt daily [23]. Moreover, urinary proteins decreased more in patients with low-or-medium sodium diet than in those in high-sodium intake group. The excess risk of ESRD was independent of blood pressure control and was largely explained by the blunted antiproteinuric effect of ACEi therapy in the setting of sodium overload [23]. Consequently, a diet containing an excess of sodium chloride, like that of diets with unregulated phosphate intake, can blunt the antiproteinuric effect of ACEi while increasing the risk for developing ESRD.

## Dietary changes ameliorate mineral bone disorders of CKD

Chronic kidney disease-mineral and bone disorder (CKD-MBD) is a systemic dysfunction of mineral and bone metabolism in patients with CKD. It results from abnormalities in calcium, phosphorous, parathyroid hormone and/or vitamin D metabolism as well as abnormalities in bone turnover, mineralization, volume, linear growth or strength, in addition to vascular or other soft tissue calcification [24]. Phosphate retention plays a crucial role in the development of CKD-MBD and it also increases the risk of cardiovascular events and mortality in patients with CKD [25, 26]. In the early stages of CKD serum phosphate levels are maintained in the normal range through phosphaturia induced by increase in parathyroid hormone actions and fibroblast growth factor-23 (FGF-23) production. However, as kidney disease progresses the compensatory rise in FGF-23 levels fails to sustain phosphate clearance sufficiently and hyperphosphatemia ensues. Notably, increased FGF-23 levels by itself has been associated with increases in both cardiovascular events and mortality [27, 28], suggesting that control of phosphate homeostasis early in CKD may help reducing the clinical consequences of mineral and bone disorders. Strategies to manage elevated serum phosphorus levels include reduction of dietary phosphate intake, as well as the use of phosphate binding agents. Clinical studies have assessed the effects of dietary phosphate and protein restriction in CKD patients. For example, patients with advanced CKD (stages 4 and 5) who had a reduction in dietary phosphates and protein intake also had improved short-term control of secondary hyperparathyroidism [29–32]. In the long term, altering the diet allowed some patients to achieve a normal rate of bone formation [33]. Recent evidence suggests that in addition to the absolute amount of phosphate in the diet, its source (i.e., plant *versus* animal food) should be considered [34]. Indeed, dietary phosphates from plant-derived proteins are mostly in the form of phytates, which are less digestible in humans. Consequently, phosphates are ultimately less bioavailable compared to phosphates derived from animal sources [34]. Moe et al*.* addressed this issue in nine patients who had established CKD (mean eGFR 32 mL/min/1.73 m^2^) by comparing their responses to vegetarian- or meat-based diets with equivalent contents of protein and phosphates. After one week of the dietary intervention in their crossover study, the vegetarian diet led to lower serum levels of phosphorus and FGF-23 than the meat-based diet [35]. Moreover, 13 patients with stages 3–5 CKD converted their food choices from an animal protein-based diet to a 70 % plant-protein diet for four weeks. The result was a significant reduction of urinary phosphorous excretion [36] even though there were no significant changes in serum phosphorous or FGF-23 levels. The urinary sodium and titratable acid contents significantly decreased with the change to plant-based foods [36]*.* Overall, the combination of phosphate-restricted diets plus oral phosphate binders has become a well established approach to controlling serum phosphorus levels in patients with CKD stages 3–5 (including CKD stage 5D) [24]. For example, a randomized controlled trial showed that dual intervention with a low-phosphorous diet and the addition of phosphate binders was more effective than either approach alone in reducing serum FGF-23 concentrations in 39 patients with CKD stages 3–4 and normal serum phosphate levels [37]. Taken together, the results we have discussed suggest that restriction of dietary phosphate intake may improve the control of mineral and bone metabolism in CKD patients. Nevertheless, the prescription of low phosphate diets can be challenging in that the bioavailability of phosphates in foods (see above) needs to be considered.

## Is the low protein diet-ketoacid regimen nutritionally adequate?

The MDRD Study of nondiabetic patients with stage 4 CKD, was designed to examine the effects of restricted dietary protein and blood pressure control on the rate of decline in measured GFR in 255 patients [2]. During approximately 2 years of study, patients were treated with either a low-protein (0.58 g/kg/day) diet or a very low-protein diet (0.28 g/kg/day) that was supplemented with a special mixture of essential keto acids and amino acids (KA regimen). Adverse outcomes associated with the diets included the development of kidney failure (initiation of dialysis therapy or transplantation), death or a composite outcome of kidney failure or death. It was concluded that the frequencies of these outcomes between the two dietary regimens were scarcely different; there was only a slightly slower decline in GFR associated with the very low protein-KA regimen. Following the announcement of this conclusion, the manufacturers of the essential ketoacid-amino acid mixture stopped their production of ketoacids and amino acids; the availability of ketoacids in the U.S. ceased and patients were encouraged to design their own diets. Over the ensuing 10 years after the MDRD Study ended, data regarding dietary changes, nutritional measurements and the development of other diseases were not evaluated systematically. Consequently, it is difficult to evaluate the report that during the 10 years following the end of the MDRD Study, the frequency of death in patients treated with the ketoacid regimen was higher than the rate occurring in patients assigned to the low protein, non-supplemented diet [38]. Specifically, insights or conclusions about death rates that occurred following participation in the MDRD trial are not possible because there were no records of differences in compliance with the dietary regimens during the MDRD trial or types of diets after cessation of the MDRD trial, types of renal replacement therapy, variability in health status, drug treatment regimens, etc. during the years following the end of the MDRD Study. Still, the report did stimulate other investigators to examine the efficacy and toxicity of the very low protein, KA regimen. Chauveau and colleagues examined the long-term outcomes of patients with CKD stage 4–5 who were treated with a ketoacid-supplemented very low protein diet [39]. Among the 251 patients they enrolled, approximately 8.4 % discontinued the diet regimen within less than 3 months. The final analysis focused on 203 patients who were given the dietary treatment for more than 3 months (mean duration 3 years) before starting renal replacement therapy. The investigators reported that the survival rates in the study population receiving renal replacement therapy after stopping treatment with KA-supplemented very low protein diet were 79 % and 63 % at 5 and 10 years, respectively [39]. There was no evidence that the KA regimen jeopardized the survival of 102 patients who were subsequently treated by chronic dialysis nor was there reduced survival of 101 patients who received a kidney transplant. In fact, they reported there was no correlation between death rates in patients treated by the KA regimen and the duration of treatment with the KA regimen. Why the outcomes between investigators in France [39] and the Menon [38] report are so different is not known but the paucity of information about events, illnesses, etc. of MDRD patients treated with the KA regimen indicates we are unlikely to obtain the information needed to identify explanation(s) for the differences. Nevertheless, the report by Chauveau and colleagues provides evidence that long term ketoacid-based dietary regimens are nutritionally sound, at least in those who were compliant to the therapy [39, 40]. Moreover, there is reassuring, albeit limited, information about the utility of dietary treatment in patients who are at increased risk of adverse outcomes, namely, elderly individuals with advanced renal insufficiency.

Brunori and colleagues examined the outcomes of different treatment regimens for elderly (≥70 years) patients without diabetes but with advanced renal insufficiency (i.e., eGFRs of 5 to 7 mL/min.) [41]. In their report, patients were randomly assigned to either a very low protein, vegan diet that was supplemented with ketoacids (56 patients) or to dialysis without dietary intervention (56 patients). Over the median treatment period of 26.5 months, the death rates of the two groups of elderly patients were not statistically different nor were the survival rates obtained following one year of the therapies when the outcomes were subjected to an intention-to-treat analysis. The authors concluded that the very low protein diet supplemented with ketoacids was a safe method for postponing dialysis treatment for non-diabetic, elderly patients. Notably, these outcomes were examined by a cost-benefit analysis [42]. It was estimated that each patient treated with the low dietary protein, KA regimen diet saved the National Health Service 21,180 euros per patient when dialysis was postponed for 1 year.

The most recent analysis of responses to dietary modification and the efficacy of the very low protein diet supplemented by the ketoacids was reported by Garneata et al. [43]. These investigators conducted a prospective, randomized controlled trial to assess the safety and efficacy of a ketoacid-supplemented very low protein regimen (0.3 g dietary protein/kg/day) compared to a conventional low protein diet (0.6 g dietary protein/kg/day) in non-diabetic patients with <30 ml/min/1.73 m^2^ GFR and < 1 g urinary protein/g creatinine. The trial was designed to test responses to the very low protein diet-KA regimen rigorously because the investigators tested patients for dietary compliance and only enrolled patients who demonstrated compliance. As expected, achieving the desired characteristics for enrolling patients required screening of a very large number of individuals (more than 1400) between March, 2006 and April, 2009. Thus, the trial encompassed 104 patients treated with the KA regimen and 103 patients treated with the conventional low protein diet. Both types of nutritional interventions were safe, as reflected by stable anthropometric and biochemical parameters (e.g., body mass index, serum albumin, or estimates of muscle mass). Regarding efficacy in avoiding end-stage renal disease, evidence from the intention-to-treat analysis led the authors to conclude that 55 patients reached the primary composited efficacy end point (i.e., initiation of dialysis or a >50 % reduction in eGFR). Importantly, only 13 % of patients in the KA regimen group reached the pre-specified end points while 42 % in the conventional low protein diet group reached the end points (*P* < 0.001). These outcomes were also used to calculate the adjusted number of patients needed to treat with the very low protein diet KA regimen for 1 year to avoid reaching the composite endpoint, that was only 4.4 CKD patients. Notably, it was determined that the serum bicarbonate levels for patients treated with the KA regimen averaged 23 mM; compared to only 16 mM for patients treated with the conventional low protein diet. The authors concluded that the very low protein diet KA regimen is nutritionally safe and could defer for almost 1 year the need for dialysis in patients with eGFR <30 ml/min/1.73 m^2^ [43]. Although the clinical outcome achieved with the very low protein diet KA regimen was significant, the participants were relatively well-selected and motivated underscoring the challenge for investigators to uncover methods of improving compliance and efficacy of diets for patients with CKD. The low enrollment rate of the Garneata trial [43] may also suggest that for CKD patients the choice of low-protein diet regimen or dialysis are not equivalent. This possibility could be tested in observational studies where patients will be assigned to low-protein or conventional diet according to their will.

Why is dietary counseling used so infrequently? We believe there are four major reasons: 1) designing the diets is complex and requires a skilled dietician which can be costly (in the U.S., this cost is paid for Medicare recipients). 2) Changing the diet can be complicated when there are other illnesses. 3) There is concern that low-protein diet could cause loss of protein stores or other complications. 4) The outcomes from the MDRD trial led to the conclusion that low-protein diets did not significantly slow progression in non-diabetic, CKD patients.

## New strategies to improve lean body mass, a complication of CKD

In patients with CKD, muscle wasting and limited physical performance can substantially contribute to reduced confidence and the risk of injuries. This is especially true for patients who are bedridden or immobile [44]. For example, measurements of the physical status of 385 patients with stages 2–4 CKD, uncovered a reduction in muscle mass plus variabilities in the functional abilities of different muscles [45]. The authors concluded that patients with CKD were characterized by leg performances that were at least 30 % lower than predicted while handgrip strength was relatively preserved. The major abnormality in muscle functioning was a reduction in measurements of walking speed and the “timed and up and go” testing. The presence of these abnormalities predicted a 3-year risk of death, was more reliably than variations in the degree of CKD or the presence of other biomarkers, and when eGFR levels were combined with measurements of walking, the mortality predictions were more accurate. In summary, impaired physical performance of the lower extremities is common in CKD and strongly associates with all-cause mortality.

The results we have highlighted raise the questions whether it is possible to prevent loss of muscle mass and would success in this goal improve the physical performance of patients with CKD to ultimately reduce their risks of mortality? Devising methods that prevent muscle loss has long been a goal of the treatment of patients with CKD. As noted earlier, correction of metabolic acidosis not only prevents muscle wasting in animal-based models of CKD but also improves nutritional indices of patients with CKD and metabolic acidosis [4, 6]. The mechanisms underlying the loss of muscle mass in rodent models of CKD (and other catabolic conditions) have been closely linked to the development of insulin resistance, the generation of inflammation and the activation of proteolysis in the ubiquitin-proteasome [19, 46–49]. Recently, methods have been designed that can suppress the protein wasting stimulated by insulin resistance and/or inflammation. For example, it is possible to block inflammation and muscle wasting that develops in mice with CKD by inhibiting myostatin [47]. Similarly, blockade of Stat3 has been found to suppress both muscle wasting and the generation of inflammatory cytokines, suggesting that these inhibitors may have transitional relevance [48]. We recognize that these are only the first steps in the goal of devising a method that safely improves muscle mass and obviously, this will require intensive testing in patients with catabolic conditions, including CKD.

## Conclusions

We conclude that integrating dietary manipulations into a comprehensive strategy will help prevent or ameliorate complications of CKD including acidosis, hyperkalemia, hyperphosphatemia and uremic symptoms and possibly influence CKD progression. We believe particular attention should be paid: 1) to correcting metabolic acidosis with either sodium bicarbonate supplements or more simply with diet instructions how to include supplements of fruits and vegetables; and 2) to lower the intake of sodium chloride as well as phosphates by choosing foods that provide low contents of these ions. Changing the diet by concentrating on these dietary constituents will allow us to maximize the renoprotective anti-proteinuric effect of renin angiotensin system blockers. These considerations on dietary approaches for CKD prevention and management are particularly valuable for low-resource setting worldwide where patients with CKD are beset with numerous challenges often traceable to poverty and a lack of access to life-saving dialysis and transplantation [50]. The feasibility of these management approaches for CKD and its risk factors even in low-income countries is exemplified by the program set up in the communities of Eastern Nepal in collaboration with the International Society of Nephrology (ISN) [51]. Dietary recommendation of low-sodium intake and increase of fruits and vegetable together with low-cost anti-hypertensive, anti-diabetic or renoprotective drugs as deemed appropriate, have been able to control proteinuria and slow renal function decline in more than a third of 3,400 individuals on active monitoring.

Regarding the very low protein diet with essential keto acids and amino acids regimen, there is recent and reassuring evidence for its efficacy and nutritionally safety but efforts are needed to improve compliance with dietary regimens. Nutritional educational programs and dieticians could help to increase patient adherence to dietary recommendations. We also recommend that components of the diet and regular monitoring of nutritional status should be jointly assessed by physicians and dieticians, just as recommended for patients with inherited metabolic defects, cirrhosis and diabetes.
